# Early LPS-induced ERK activation in retinal pigment epithelium cells is dependent on PIP_**2**_**-**PLC^☆^

**DOI:** 10.1016/j.dib.2016.02.057

**Published:** 2016-02-27

**Authors:** Melina V. Mateos, Constanza B. Kamerbeek, Norma M. Giusto, Gabriela A. Salvador

**Affiliations:** Instituto de Investigaciones Bioquímicas de Bahía Blanca (INIBIBB), Universidad Nacional del Sur (UNS) and Consejo Nacional de Investigaciones Científicas y Técnicas (CONICET), Camino La Carrindanga km 7, 8000 Bahía Blanca, Argentina

**Keywords:** ERK, extracellular signal-regulated kinase, HRP, horseradish peroxidase, LPS, lipopolysaccharide, PIP_2_-PLC, phosphatidylinositol bisphosphate-phospholipase C, PLD, phospholipase D, RPE, retinal pigment epithelium, Retinal pigment epithelium (RPE), Inflammation, Phosphatidylinositol bisphosphate-phospholipase C (PIP_2_-PLC), Phospholipase D (PLD), Extracellular signal-regulated kinase (ERK1/2), Lipopolysaccharide (LPS)

## Abstract

This article presents additional data regarding the study “The phospholipase D pathway mediates the inflammatory response of the retinal pigment epithelium” [1]. The new data presented here show that short exposure of RPE cells to lipopolysaccharide (LPS) induces an early and transient activation of the extracellular signal-regulated kinase (ERK1/2). This early ERK1/2 activation is dependent on phosphatidylinositol bisphosphate-phospholipase C (PIP_2_-PLC). On the contrary, neither the phospholipase D 1 (PLD1) nor the PLD2 inhibition is able to modulate the early ERK1/2 activation induced by LPS in RPE cells.

## **Specifications table**

**Table:** 

Subject area	*Biochemistry*
More specific subject area	*Cell biology*
Type of data	*WB images, bar graphs*
How data was acquired	*Western blot. Densitometry values were obtained using the ImageJ software*
Data format	*Raw and analyzed*
Experimental factors	*ARPE-19 cells were exposed to LPS. Pharmacologycal inhibitors of PLD1, PLD2 and PIP*_*2*_*-PLC were used.*
Experimental features	*ERK1/2 activation was evaluated by Western blot*
Data source location	*Instituto de Investigaciones Bioquímicas de Bahía Blanca (INIBIBB), Universidad Nacional del Sur (UNS) and Consejo Nacional de Investigaciones Científicas y Técnicas (CONICET), 8000 Bahía Blanca, Argentina.*
Data accessibility	*Data is provided within the article*

## **Value of the data**

• The data can be useful to other scientists investigating the effects of LPS on RPE cells.
• The data provide additional information regarding the LPS-induced ERK1/2 signaling in RPE cells.
• Results shown here demonstrate that the early and the late LPS-induced ERK1/2 activation are differentially modulated by PIP2-PLC and PLD pathways.

## 1. Data

The data presented here show that in RPE cells, ERK1/2 activation induced by 5 min treatment with LPS depends on PIP_2_-PLC but is not affected by classical PLDs inhibition.

## 2. Experimental design, materials and methods

### 2.1. Retinal-pigmented epithelium cell culture and treatments

Human retinal-pigmented epithelium cells (ARPE-19) were maintained in Dulbecco’s Modified Eagle’s Medium (DMEM) supplemented with 10% fetal bovine serum (FBS, Natocor, Argentina), 100 U/ml penicillin, 100 μg/ml streptomycin and 0.25 μg/ml amphotericin B at 37 ˚C under 5% CO_2_. Confluent 35 mm diameter cell dishes were serum-starved for 2 h prior to stimulation for 5 min or 2 h with 10 μg/ml of *Pseudomonas aeruginosa* LPS in serum-free DMEM. Sterile ultra pure water was added to the control condition. In order to inhibit PIP_2_-PLC and PLDs pathways ARPE-19 cells were preincubated with selective inhibitors for 1 h at 37 °C prior to cell stimulation with LPS. 0.15 μM EVJ was used to inhibit PLD1 activity and 0.5 μM APV to inhibit PLD2. For PIP_2_-PLC inhibition, cells were preincubated with U73122 (10 μM). DMSO (vehicle of the inhibitors) was added to all conditions to achieve a final concentration of 0.025% [1], [2].

### 2.2. Western blot analysis (WBs)

After experimental treatment the medium was removed, cells were washed three times with PBS and scraped off with 80 μl ice-cold RIPA lyses buffer (10 mM Tris–HCl (pH 7.4), 15 mM NaCl, 1% Triton X-100, 5 mM NaF, 1 mM Na_2_VO_4_ and the complete protease inhibitor cocktail). Protein content of cellular lysates was determined by Bradford method [3] (Bio-Rad Life Science group, #500-0006). Samples were denatured with Laemmli sample buffer at 100 °C for 5 min [4]. Equivalent amounts of proteins (30 μg) were separated by sodium dodecyl sulfatepolyacrylamide gel electrophoresis (SDS-PAGE) on 10% polyacrylamide gels, transferred to polyvinylidene fluoride (PVDF) membranes (Millipore, Bedford, MA, USA) and WBs were performed as previously described [1]. Rabbit polyclonal antibody anti-phospho-ERK1/2 (#9101) was from Cell Signaling (Beverly, MA, USA). Mouse monoclonal anti-α Tubulin (DM1-A) (CP06) was from EMD/Biosciences-Calbiochem (San Diego, CA, USA). HRP-conjugated goat anti-rabbit IgG and HRP-conjugated goat anti-mouse IgG were purchased from Santa Cruz Biotechnology, Inc. (Santa Cruz, CA, USA). Densitometry values of the immunoreactive bands were determined using ImageJ 1.38 software.

### 2.3. Statistical analysis

Statistical analysis was performed using ANOVA followed by Bonferroni׳s test to compare means. *p-*Values lower than 0.05 were considered statistically significant. Data represent the mean value±SD of at least three independent experiments. The WBs shown are a representative image of samples from at least three independent experiments.

## 3. Results

**Fig. 1:**
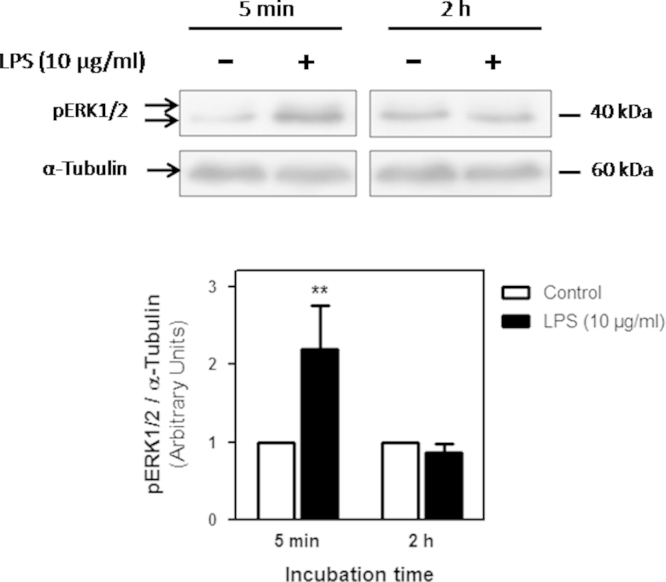
ERK1/2 activation in ARPE-19 cells exposed to LPS. ARPE-19 cells were treated with LPS (10 µg/ml) or ultra pure water (control condition) for 5 min or 2 h. ERK1/2 activation was evaluated by WB assays using anti-phospho ERK1/2 (pERK1/2) antibody. Numbers to the right indicate molecular weights and the bar graph shows the densitometry values of pERK1/2/ α-Tubulin expressed as arbitrary units as ratio of the control. Asterisks indicate significant differences with respect to the control condition (**p<0.01).

**Fig. 2:**
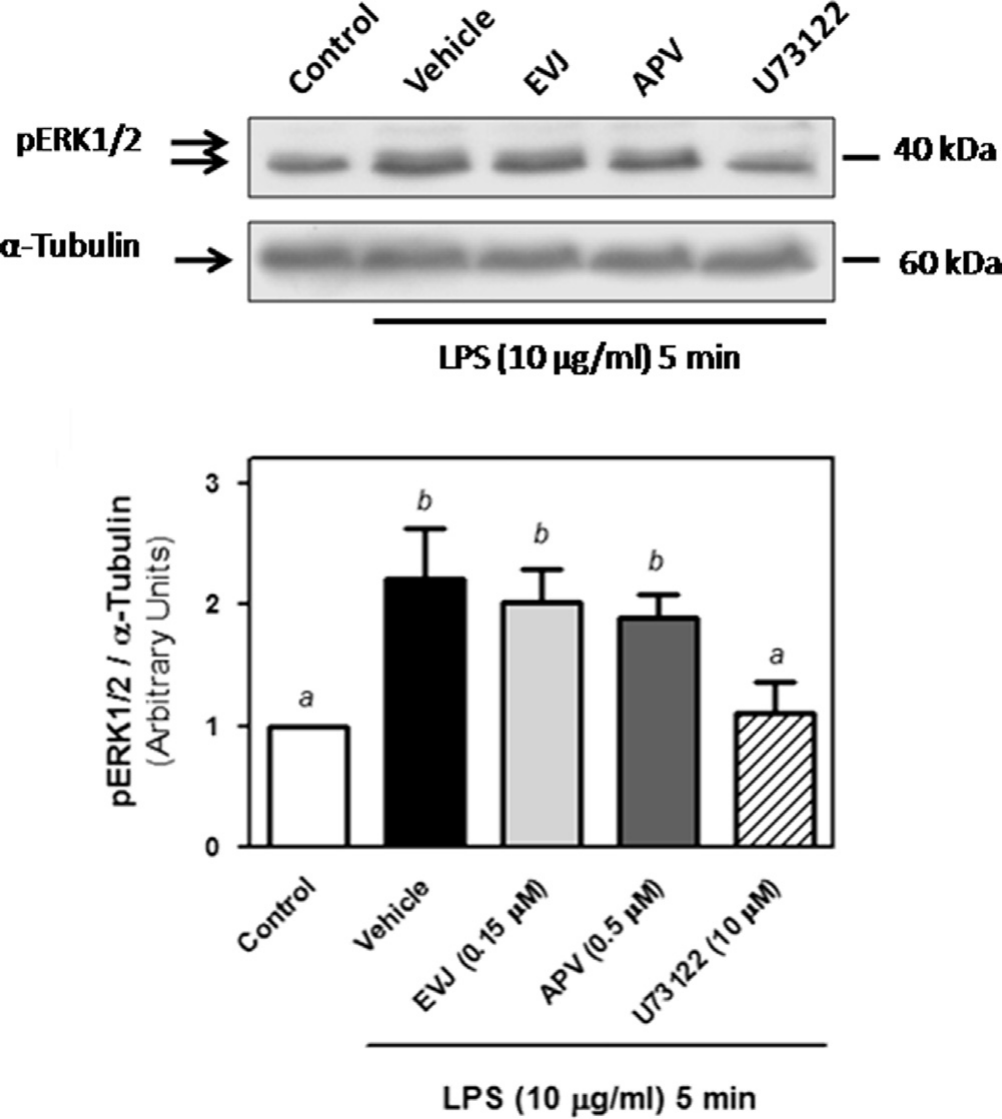
Role of PIP_2_-PLC and PLDs in ERK1/2 activation. ARPE-19 cells were preincubated with 0.025% DMSO (control and vehicle conditions), 0.15 µM EVJ, 0.5 µM APV or 10 µM U73122 for 1 h before LPS treatment. Cells were treated with 10 µg/ml LPS or ultra pure water (control condition) for 5 min. Numbers to the right indicate molecular weights and the bar graph shows the densitometry values of pERK1/2/ α-Tubulin expressed as arbitrary units as ratio of the control. (a and b) Indicate significant differences among columns (*p*<0.05).

As shown in Fig. 1, 5 min exposure to LPS (10 µg/ml) induced a strong activation of ERK1/2 (120% compared to the control condition) in ARPE-19 cells. This activation is transient since it is no longer observed after 2 h treatment with LPS. The transient ERK1/2 activation observed after 5 min stimulation with LPS was only prevented by the PIP_2_-PLC inhibitor U73122 but was not affected by PLD1 or PLD2 inhibition (Fig. 2). These data demonstrate that the LPS-induced early ERK1/2 depends exclusively on PIP_2_-PLC (Fig. 2), while PLD2 activation is necessary to maintain ERK1/2 phosphorylation after 24 h treatment [1].

## References

[1] Mateos M.V., Kamerbeek C.B., Giusto N.M., Salvador G.A. (2014). The phospholipase D pathway mediates the inflammatory response of the retinal pigment epithelium. Int. J. Biochem. Cell. Biol..

[2] Mateos M.V., Giusto N.M., Salvador G.A. (1823). Distinctive roles of PLD signaling elicited by oxidative stress in synaptic endings from adult and aged rats. Biochim. Biophys. Acta.

[3] Bradford M.M. (1976). A rapid and sensitive method for the quantitation of microgram quantities of protein utilizing the principle of protein-dye binding. Anal. Biochem..

[4] Laemmli U.K. (1970). Cleavage of structural proteins during the assembly of the head of bacteriophage T4. Nature.

