# Supplementary material for: Early LPS-induced ERK activation in retinal pigment epithelium cells is dependent on PIP2-PLC
Source: Data Brief. 2016 Feb 27;7:423–7. doi: 10.1016/j.dib.2016.02.057 (PMC4786752; doi:10.1016/j.dib.2016.02.057)
Supplement: Supplementary file 1 — Supplementary material [file mmc1.docx]

*Data article (DIB-D-15-00680)*

**Early LPS-induced ERK activation in retinal pigment epithelium cells is dependent on PIP_2_-PLC**

Melina V. Mateos, Constanza B. Kamerbeek, Norma M. Giusto and Gabriela A. Salvador

**The authors declare that there is not conflict of interest.**
